# Supplementary material for: Disease progression role as well as the diagnostic and prognostic value of microRNA-21 in patients with cervical cancer: A systematic review and meta-analysis
Source: PLoS One. 2022 Jul 27;17(7):e0268480. doi: 10.1371/journal.pone.0268480 (PMC9328569; doi:10.1371/journal.pone.0268480)
Supplement: S2 Table — Studies were searched from databases using key terms. (DOCX) [file pone.0268480.s002.docx]

| **Database searched** | **Terms and keywords used** | Filtering description | Results | Total result |
| --- | --- | --- | --- | --- |
| PubMed/MEDLINE | microRNA 21 AND cervical cancer AND diagnosis | All fields | 65 | 563 |
|  | microRNA 21 AND cervical cancer AND Prognosis | All fields | 40 |  |
|  | microRNA 21 AND cervical cancer AND Target | All fields | 89 |  |
|  | ((((microrna[MeSH Terms]) OR (micrornas[MeSH Terms])) AND (uterine cervical cancer[MeSH Terms])) AND (cancer early diagnosis[MeSH Terms])) NOT (book reviews[MeSH Terms]) | Mesh terms advanced search | 28 |  |
|  | ((micrornas[MeSH Terms]) AND (cancer, uterine cervical[MeSH Terms])) AND (prognosis[MeSH Terms]) | Mesh terms advanced search | 222 |  |
|  | ((micrornaS[MeSH Terms]) AND (cancer, uterine cervical[MeSH Terms])) AND (disease progression[MeSH Terms]) | Mesh terms advanced search | 119 |  |
| WorldCat  (Advanced search) | kw:miR-21 AND cervical cancer AND diagnosis | Downloadable articles published in English | 19 | 74 |
|  | kw:miR-21 AND cervical cancer AND prognosis | Downloadable articles published in English | 11 |  |
|  | kw:miR-21 AND cervical cancer AND target | Downloadable articles published in English | 44 |  |
| DOAJ | miR-21 AND cervical cancer AND diagnosis | All fields | 13 | 32 |
|  | miR-21 AND cervical cancer AND prognosis | All fields | 6 |  |
|  | miR-21 AND cervical cancer AND progression | All fields | 13 |  |
| ScienceDirect  (Advanced search) | MicroRNA-21 Cervical cancer Diagnosis | Limited to research articles article type, 2 publication title and 2 subject areas | 105 | 302 |
|  | MicroRNA-21 Cervical cancer prognosis | Limited to research articles article type, 1 publication title and 1 subject area | 120 |  |
|  | MicroRNA-21 "Cervical cancer" "disease progression" | Limited to research articles article type and 2 subject areas | 77 |  |
| Google Scholar (advanced search) | microRNA-21 OR miR-21 "cervical cancer" "diagnos OR prognos" | All | 160 | 174 |
|  | allintitle: microRNA 21 "cervical cancer" | All | 14 |  |
| Web of Science | microRNA-21 (Topic) or microRNA-21 (Title) or microRNA-21 (Abstract) or miRNA-21 (Topic) or miRNA-21 (Title) or miRNA-21 (Abstract) or miR-21 (Topic) or miR-21 (Title) or miR-21 (Abstract) or microRNA 21 (Topic) or microRNA 21 (Title) or microRNA 21 (Abstract) or miRNA 21 (Topic) or miRNA 21 (Title) or miRNA 21 (Abstract) or miR-21-5p (Topic) or miR-21-5p (Title) or miR-21-5p (Abstract) or miR-21-3p (Topic) or miR-21-3p (Title) or miR-21-3p (Abstract) AND Cervical cancer (Topic) or cervical neoplasm (Title) or Cervical cancer (Abstract) or Cervical neoplasm (Topic) or cervical neoplasm (Abstract) or cervical tumor (Topic) or cervical tumor (Title) or cervical tumor (Abstract) AND Diagnosis (Topic) or Diagnosis (Title) or Diagnosis (Abstract) or Prognosis (Topic) or Prognosis (Title) or Prognosis (Abstract) |  |  | 112 |
| EMBASE | 'uterine cervix cancer':ti,ab,kw OR 'cervical neoplasm':ti,ab,kw OR 'cervical tumor':ti,ab,kw OR 'uterine cervix cancer'/exp AND 'microrna 21':ti,ab,kw OR 'mir 21':ti,ab,kw OR 'mirna 21':ti,ab,kw OR 'mir 21 5p':ti,ab,kw OR 'mir 21 3p':ti,ab,kw OR 'microrna 21'/exp AND diagnosis:ti,ab,kw OR 'diagnostic procedure':ti,ab,kw OR prognosis:ti,ab,kw OR 'diagnosis'/exp OR 'prognosis'/exp |  |  | 105 |

S2 Table. Articles searching history from different databases. Studies were searched from databases using key terms.
