# Supplementary material for: Disease progression role as well as the diagnostic and prognostic value of microRNA-21 in patients with cervical cancer: A systematic review and meta-analysis
Source: PLoS One. 2022 Jul 27;17(7):e0268480. doi: 10.1371/journal.pone.0268480 (PMC9328569; doi:10.1371/journal.pone.0268480)
Supplement: S3 Table — New Castle Ottawa Scale was used for prognostic component. (DOCX) [file pone.0268480.s003.docx]

| **S3 Table. Quality evaluation result of disease progression role of microRNA-21 component.** | | | | | | | | | |
| --- | --- | --- | --- | --- | --- | --- | --- | --- | --- |
| **No** | **Author (publication year)** | **Methos for Expression of microRNA** | **Total Sample** | **Cancer cell line** | **Target** | | | | **Scimgo journal ranking for the articles** |
|  | Wilting et al 2013 | microarray and RT-PCR | 47 | SiHa cell line | Effect on Cervical carcinogenesis | | | | Q1 |
|  | Deftereos et al 2011 | Taqman qPCR | 72 | SiHa, CaSki, HeLa | Cancer invasiveness and PDCD4, PTEN and TPM1 expression | | | | Q1 |
|  | Shishodia et al 2015 | RT-PCR and immunoblotting | 102 | C33a (HPV-negative), CaSki and SiHa (HPV16-positive) and HeLa (HPV18-positive) | Cervical carcinogenesis | | | | Q1 |
|  | Bumrungthai et al 2015 | RT-PCR and MicroRNA in situ hybridization | 181 | HeLa cell line | Inflammation process and cervical cancer progression | | | | Q1 |
|  | Zeng et al 2015 | Microarray and RT-PCR | 103 | HeLa cells | Cancer cell invasion and migration | | | | Q2 |
|  | Tang et al 2020 | RT-PCR | 45 | HeLa and SiHa cells | Cancer cell metastasis | | | | Q3 |
|  | McBee et al 2011 | Microarray and RT-PCR | 13 | - | Invasiveness of cervical cancer | | | | - |
|  | Xue et al 2016 | RT-PCR | 80 | A549 and H1299 | Downregulating SOCS1, SOCS6, and PTEN | | | | Q2 |
|  | Liwak-Muir et al 2016 | RT-PCR | - | HeLa cells | Down regulating PDCD4 | | | | Q2 |
|  | Balacescu et al 2017 | - | - | - | BCL2↓, PTEN↓, PDCD4↓, BTG2↓, TPM1↓ | | | | - |
|  | Xu et al 2017 | RT-PCR | - | HeLa cells | TNF-α↓ | | | | Q3 |
|  | Yang et al 2016 | - | - | - | NF-κB signaling pathway | | | | Q2 |
|  | Arcaro 2014 | - | - | - | PI3K/mTOR signaling | | | | Q1 |
|  | Porta et al 2014 | - | - | - | PI3K/mTOR signaling | | | | Q1 |
|  | Chen et al 2015 | Sequencing and qRT-PCR | - | HeLa cells | PTEN | | | | Q1 |
|  | Li et al 2018 |  | - | SKM-1, SH-SY5Y, SRA01/04 and Kasumi-1 cell lines | PTEN/AKT pathway | | | | Q3 |
|  | Peralta-Zaragoza et al 2016 | RT-PCR | - | SiHa and HeLa | PTEN pathway | | | | Q2 |
|  | Liu et al 2013 | qRT-PCR | - | Eca109 | ERK1/2/MAPK pathway | | | | Q2 |
|  | Gong et al 2015 | qRT-PCR | - | RKO, HCT116, colo205, SW480, HT29, and Caco2 | RAS pathways | | | | Q1 |
|  | Zhang et al 2016 | qRT-PCR | 109 | HeLa and  HT-3 | RasA1 | | | | Q2 |
|  | Tidyman et al 2009 | - | - | - | RAS/RAF/MEK/ERK | | | | Q1 |
|  | Hatley et al 2010 | Northern blot  analysis and real-time PCR | - | Mice model | Ras/ MEK/ERK | | | | Q1 |
|  | Aksamitiene et al 2012 | - | - | - | Ras/MAPK and PI3K/Akt cross-talk | | | | Q1 |
|  | Martin et al 2015 | qRT-PCR | - | WM1552c, WM793b | TIMP3/ MMPs | | | | Q1 |
|  | Nagao et al 2012 | RT-PCR and Microarray | 65 | Tissue biopsy | TIMP3 | | | | Q1 |
|  | Zhang et al 2018 | qRT-PCR | 120 | Tissue biopsy | TIMP3 | | | | Q2 |
|  | Matsuhashi et al 2019 | - | - | - | PDCD4 | | | | Q1 |
|  | Yao et al 2009 | qRT-PCR | - | HeLa cells | PDCD4 | | | | Q1 |
|  | Yang et al 2003 | qRT-PCR | - | - | PDCD4 | | | | Q1 |
|  | Kakimoto et al 2013 | qRT-PCR | 21 | Tissue biopsy | PDCD4 | | | | Q1 |
|  | Eto et al 2012 | qRT-PCR | - | HeLa cells | PDCD4 | | | | Q1 |
|  | Yang et al 2004 | - | - | - | PDCD4 | | | | Q1 |
|  | Bae et al 2005 | - | 50 | tissue biopsies | TPM-1 | | | | Q1 |
|  | Mao et al 2015 | - | - | - | BTG2 | | | | Q2 |
|  | Buscaglia et al 2011 | - | - | - | BTG2 | | | | IF= 4.11 |
|  | Sayed et al 2010 | qRT-PCR | - | SW480, MCF-7 | FasL | | | | Q1 |
|  | Wen et al 2017 | qRT-PCR | - | Tissue and cell lines | GAS5 | | | | Q2 |
|  | Zhou et al 2020 | - | - | - | GAS5 | | | | Q3 |
|  | Cai et al 2018 | qRT-PCR | - | SiHa, HeLa, CaSki, c4-1 and c-33a cell lines and normal cervical Ect1/E6E7 cells | VHL | | | | Q3 |
|  | Yao et al 2012 | RT-PCR and Microarray | 126 | SiHa, HeLa, CaSki | CCL20 | | | | Q1 |
| **Prognostic value of microRNA-21 component** | | | | | | | | | |
|  | **Author** | **Country of study** | **Sample** | **Cancer stage** | **Sampling site** | **Testing method** | **Estimate** | **Follow up (months)** | **NOS** |
|  | Deftereos et al 2011 | Senegal | 72 | - | Tissue | qPCR | worsening histological diagnosis | - | 9 |
|  | Han et al 2015 | China | 30 | I and II | Tissue | qPCR | P<0.05 for poor prognosis parameters | - | 7 |
|  | Qiu et al 2020 | China | 202 | stage I-IIA | Serum | qRT-PCR | P=0.027 for RFS | 60 | 7 |
|  | Aftab et al 2021 | India | 170 | stage I-IV | Urine and tissue | qRT-PCR | AHR: 0.843 (0.197–3.602) | 40 | 9 |
|  | Yuan et al 2018 | China | 148 | - | Tissue | qRT-PCR | p=0.0143 survival | 60 | 8 |
|  | Hu et al 2010 | USA | 102 | stage I-IV | Tissue | qRT-PCR and microarray | p=0.02-0.05, survival rate | 40 | 9 |

**NOS**: New Castle Ottawa Scale
