# Supplementary figures and images for: Disease progression role as well as the diagnostic and prognostic value of microRNA-21 in patients with cervical cancer: A systematic review and meta-analysis
Source: PLoS One. 2022 Jul 27;17(7):e0268480. doi: 10.1371/journal.pone.0268480 (PMC9328569; doi:10.1371/journal.pone.0268480)

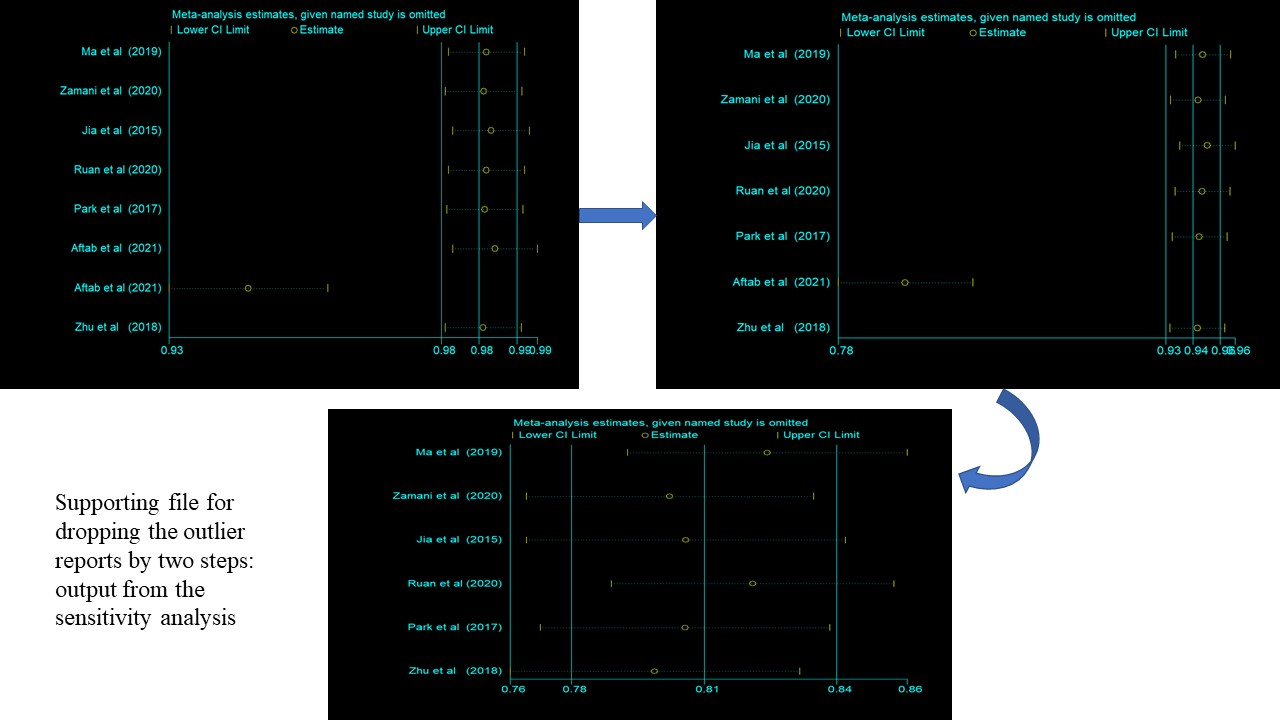

Supplement: S1 Fig — An output from sensitivity analysis. (TIF) [file pone.0268480.s004.tif]
